# Transcriptional profile of *AvrRpt2*_*EA*_-mediated resistance and susceptibility response to *Erwinia amylovora* in apple

**DOI:** 10.1038/s41598-021-88032-x

**Published:** 2021-04-22

**Authors:** Susan Schröpfer, Isabelle Vogt, Giovanni Antonio Lodovico Broggini, Andreas Dahl, Klaus Richter, Magda-Viola Hanke, Henryk Flachowsky, Andreas Peil

**Affiliations:** 1 Institute for Breeding Research on Fruit Crops, Julius Kühn Institute (JKI), Federal Research Centre for Cultivated Plants, Pillnitzer Platz 3a, 01326 Dresden, Germany; 2 Molecular Plant Breeding, Institute of Agricultural Sciences, ETH Zurich, Universitaetstrasse 2, 8092 Zurich, Switzerland; 3 DRESDEN-Concept Genome Center, Center for Molecular and Cellular Bioengineering (CMCB), Technische Universität Dresden, Fetscherstr. 105, 01307 Dresden, Germany; 4 Institute for Resistance Research and Stress Tolerance, Julius Kühn Institute (JKI), Federal Research Centre for Cultivated Plants, Erwin-Baur-Strasse 27, 06484 Quedlinburg, Germany

**Keywords:** Plant breeding, Plant genetics, Plant molecular biology, Plant stress responses

## Abstract

Most of the commercial apple cultivars are highly susceptible to fire blight, which is the most devastating bacterial disease affecting pome fruits. Resistance to fire blight is described especially in wild *Malus* accessions such as *M.* × *robusta* 5 (Mr5), but the molecular basis of host resistance response to the pathogen *Erwinia amylovora* is still largely unknown. The bacterial effector protein AvrRpt2_EA_ was found to be the key determinant of resistance response in Mr5. A wild type *E. amylovora* strain and the corresponding *avrRpt2*_*EA*_ deletion mutant were used for inoculation of Mr5 to induce resistance or susceptible response, respectively. By comparison of the transcriptome of both responses, 211 differentially expressed genes (DEGs) were identified. We found that heat-shock response including heat-shock proteins (HSPs) and heat-shock transcription factors (HSFs) are activated in apple specifically in the susceptible response, independent of *AvrRpt2*_*EA*_. Further analysis on the expression progress of 81 DEGs by high-throughput real-time qPCR resulted in the identification of genes that were activated after inoculation with *E. amylovora*. Hence, a potential role of these genes in the resistance to the pathogen is postulated, including genes coding for enzymes involved in formation of flavonoids and terpenoids, ribosome-inactivating enzymes (RIPs) and a squamosa promoter binding-like (SPL) transcription factor.

## Introduction

Fire blight, caused by the enterobacterium *Erwinia amylovora* (Burrill)^1^ is regarded as the most devastating bacterial disease affecting cultivation of pome fruit such as apple (*Malus domestica Borkh.*)^2^. The primary infection of the host by the pathogen occurs trough natural openings in flowers or wounds on vegetative tissues. Then the bacterium migrates internally to infect other organs causing blossom, shoot and rootstock blights. Fire blight outbreaks lead to significant economic losses, which are explained by lower yields, costs for pruning of infected tissue as well as eradication of entire trees or orchards^3^.

Predominate cultivars in apple production are highly susceptible to fire blight^4^, highlighting the importance of apple breeding programs to improve fire blight resistance. Genetic sources of fire blight resistance could be found in wild *Malus* species that exhibit different resistance mechanisms to combat fire blight infections^5^. Until now several quantitative trait loci (QTL) associated with fire blight resistance were identified by genetic mapping approaches. The majority of them were found in wild *Malus* accessions^5^ such as *M.* × *robusta* 5 (Mr5), *M. floribunda* 821 (Mf821), *M. arnoldiana* and *M. fusca*. In Mr5, a single resistance gene, called *FB_MR5*, located within a major QTL detected on linkage group 3 (LG3) was shown to be responsible for fire blight resistance^6,7^. *FB_MR5* belongs to the family of plant disease resistance (R) genes and encodes for a resistance protein containing a nucleotide-binding site (NBS), a C-terminal leucine rich repeat (LRR) and a coiled coil domain (CC). In general, R genes have the ability to detect a pathogen effector to initiate R-mediated host resistance response^8^ or effector-triggered immunity (ETI)^9^. The specific recognition of a pathogen is dependent on so-called effector proteins, which are delivered by the pathogen into host cells.

*E. amylovora* is known to secrete and transfer effector proteins by a type III secretion system (T3SS) into the host cytoplasm, including AvrRpt2_EA_, DspE, HopPtoC_EA_, Eop1 and Eop3 (HopX1_EA_)^10^. The dual nature of AvrRpt2_EA_, which is important for pathogenicity and resistance, was subject of several studies. The nature of AvrRpt2_EA_ acting as virulence factor on immature pear fruits was shown by Zhao et al.^11^ and its heterologous expression in the susceptible apple cultivar ‘Pinova’ led to severe fire blight symptoms^12^. In contrast, in the resistant apple genotype Mr5, AvrRpt2_EA_ acts as avirulence factor necessary for resistance response^13^. The loss of AvrRpt2_EA_ in the deletion mutant ZYRKD3-1 (Ea1189*ΔavrRpt2*_*EA*_)^11^ led to the breakdown of fire blight resistance of Mr5^13^.

Interestingly, two naturally occurring alleles of *AvrRpt2*_*EA*_ were identified in *E. amylovora* strains. The alleles differ only in one nucleotide leading to an amino acid switch (Ser/Cys), thus changing its ability to overcome fire blight resistance in Mr5^13^. *E. amylovora* strains containing the C-allele of AvrRpt2_EA_ (e.g. Ea1189) are avirulent to Mr5, whereas strains bearing the S-allele are virulent^13^. This was supported by additional studies reporting that the fire blight resistance QTL on LG3 of Mr5 is broken down by the highly aggressive Canadian strain Ea3049 containing the S-allele^14,15^. A gene-for-gene interaction in the host–pathogen system Mr5-*E. amylovora* was postulated by Vogt et al.^13^. The molecular details of AvrRpt2_EA_-recognition in the host cell are not fully elucidated, however, a direct interaction of AvrRpt2_EA_ with the R protein FB_MR5 was suggested based on analyses of the protein crystal structure of the effector^16^. Furthermore, the transgenic expression of *FB_MR5* in the fire blight susceptible cultivar 'Gala’ mediated resistance to *E. amylovora*, which was broken down by inoculation with an *avrRpt2*_*EA*_-deletion mutant strain^6^. However, the molecular mechanism behind the resistance response in this host–pathogen system is still largely unknown.

In this work, the transcriptome profiles of Mr5 inoculated with the avirulent wild type strain Ea1189 (containing the AvrRpt2_EA_ C-allele) or the virulent *avrRpt2*_*EA*_-deletion mutant strain ZYRKD3-1 were analyzed, respectively. Comparison of transcript levels between both inoculations enabled the identification of differentially expressed genes (DEGs), which belong only to the absence or presence of the effector AvrRpt2_EA_ and hence are correlated to resistant or susceptible response to *E. amylovora.* Additionally, for most DEGs potentially involved in resistant reaction, gene expression was determined by a high throughput real-time qPCR technology. The potential functions of the identified genes in relation to fire blight disease and resistance are discussed.

## Results

### RNA sequencing and mapping of the transcriptome of Mr5

To analyze the transcriptomic profile of Mr5, RNA sequencing was performed after inoculation with the avirulent wild type strain Ea1189^13^ or the virulent *avrRpt2*_*EA*_-deletion mutant of Ea1189 (ZYRKD3-1), respectively. Plant material for sequencing was collected at different time points, 2 and 48 h post infection (hpi), to include early and later response of the plant. In total, 364.572.150 reads were obtained with nearly similar distribution within the four samples (Table 1). The raw RNA-seq data has high quality as indicated by high sequence quality scores with mean values above 35. In all samples, about 50% of all obtained reads could be mapped to the reference transcriptome of *Malus domestica* cv. ‘Golden Delicious’ (GD)^17^ (Table 1), which includes crossing reads (1% per sample) and singletons (5–6% per sample), but excludes reads that mapped to more than one sites of the transcriptome (21–23% per sample).

**Table 1:** Mapping of RNA-seq data.

Read category	Ea1189				ZYRKD3-1			
	2 hpi		48 hpi		2 hpi		48 hpi	
	Reads	[%]	Reads	[%]	Reads	[%]	Reads	[%]
All obtained	81.606.040	100	115.820.750	100	73.513.950	100	93.631.410	100
Mapped to GD genome	56.686.940	69	80.589.354	70	54.303.092	74	66.824.347	71
Non uniquely mapped^1^	17.699.203	22	24.009.219	21	16.687.078	23	19.616.676	21
Cross contig^2^	817.800	1	1.203.510	1	765.520	1	1.023.886	1
Singletons^3^	5.030.528	6	6.392.451	6	4.052.216	6	5.138.139	5
Resulting reads	38.987.737	48	56.580.135	49	37.616.014	51	47.207.671	50

The transcriptome of Mr5 was sequenced at 2 and 48 hpi with the wild type strain Ea1189 and the *avrRpt2*_*EA*_ deletion mutant strain ZYRKD3-1. Numbers of reads per sample received from RNA-seq after sequencing and mapping with BWA were shown in total and percentage of all obtained reads.

^1^Number of reads mapped to more than one site of the genome, such reads were excluded from analysis. ^2^Number of reads with the other end mapped to a different contig. ^3^Number of reads with itself or its mate unmapped.

### Differential expressed genes during resistant and susceptible response

**Figure 1:**
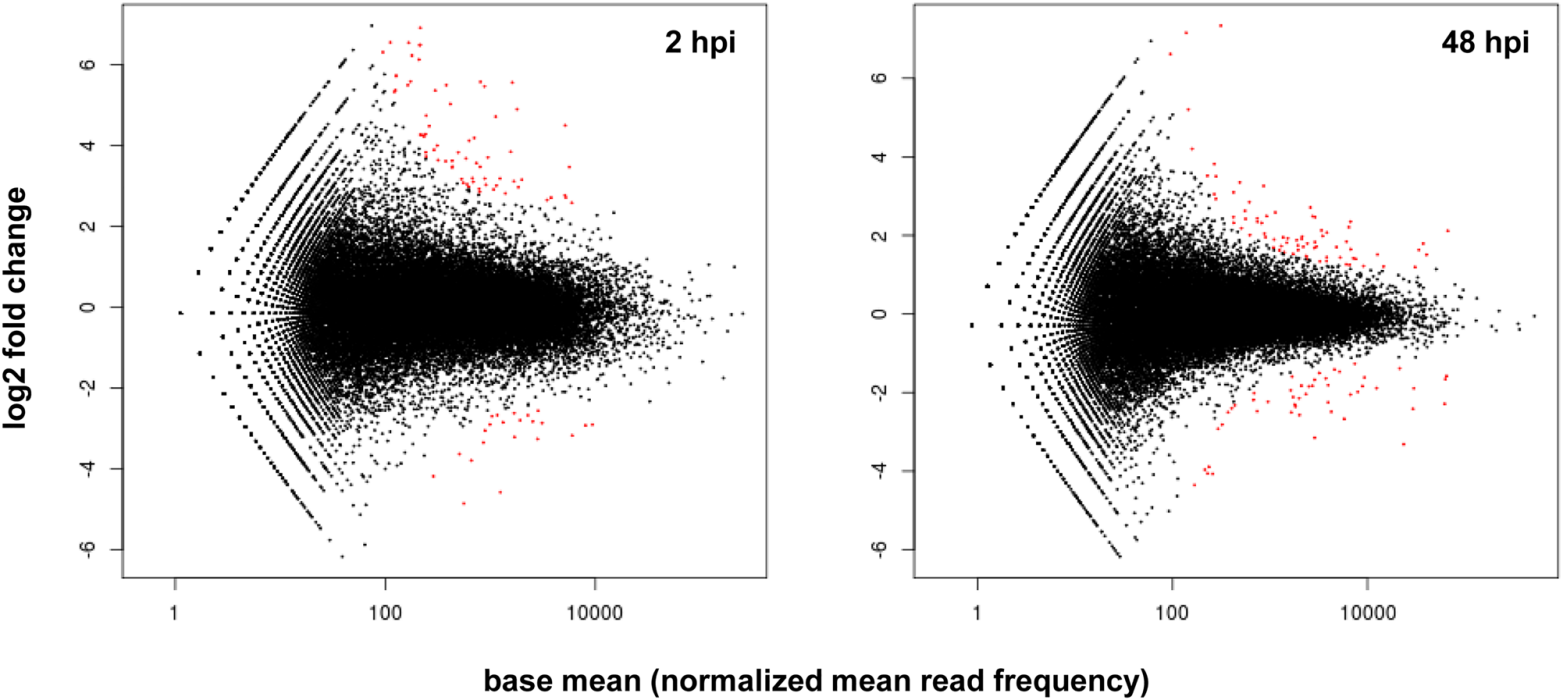
Scatter plot representing expressed transcripts. A comparison of the transcriptome of Mr5 after inoculation with the wild type strain Ea1189 and the *avrRpt2*_*EA*_ mutant strain ZYRKD3-1 was done using DESeq software package. The average of normalized read count values, dividing by size factors (base mean) is plotted against the log2 fold change at 2 hpi (left) and 48 hpi (right). Statistically significant differentially expressed genes (DEGs) are depicted as red dots (10% false discovery rate). Figure 1 was created with DESeq R package [vers. 3.0.2].

To identify DEGs, the mapped reads from the transcriptome of Mr5 challenged with the wild type strain Ea1189 (avirulent) and the *avrRpt2*_*EA*_-deleted mutant strain ZYRKD3-1 (virulent) were compared at 2 and 48 hpi. To receive an overview of the whole data set, the calculated log2 fold change of both inoculations (Ea1189 vs. ZYRKD3-1) was plotted against the normalized mean read frequency for each gene transcript (Fig. 1). Within this plot the significant DEGs are represented as red dots and identified with *p*-values less than 0.1 after they are adjusted for multiple testing with Benjamini–Hochberg correction for controlling false discovery rate. The symmetry of the plot in up- and downregulated genes was comparable between 2 and 48 hpi with a maximum log2 fold change of about 6. The analyses led to the identification of 211 DEGs, of which 85 genes showed a significant differential expression at 2 hpi, 106 genes at 48 hpi and 20 genes at both time points (Table S1). Most of these genes had a higher expression level during the resistant reaction after the inoculation of Mr5 with the wild type strain Ea1189: 83 genes at 2 hpi and 77 genes at 48 hpi, thereof 20 genes at both time points. During the susceptible reaction after inoculation of Mr5 with the *avrRpt2*_*EA*_-deleted mutant strain, 22 genes showed a higher expression level at 2 hpi and 49 genes at 48 hpi.

### Functional categorization of the DEGs

**Figure 2:**
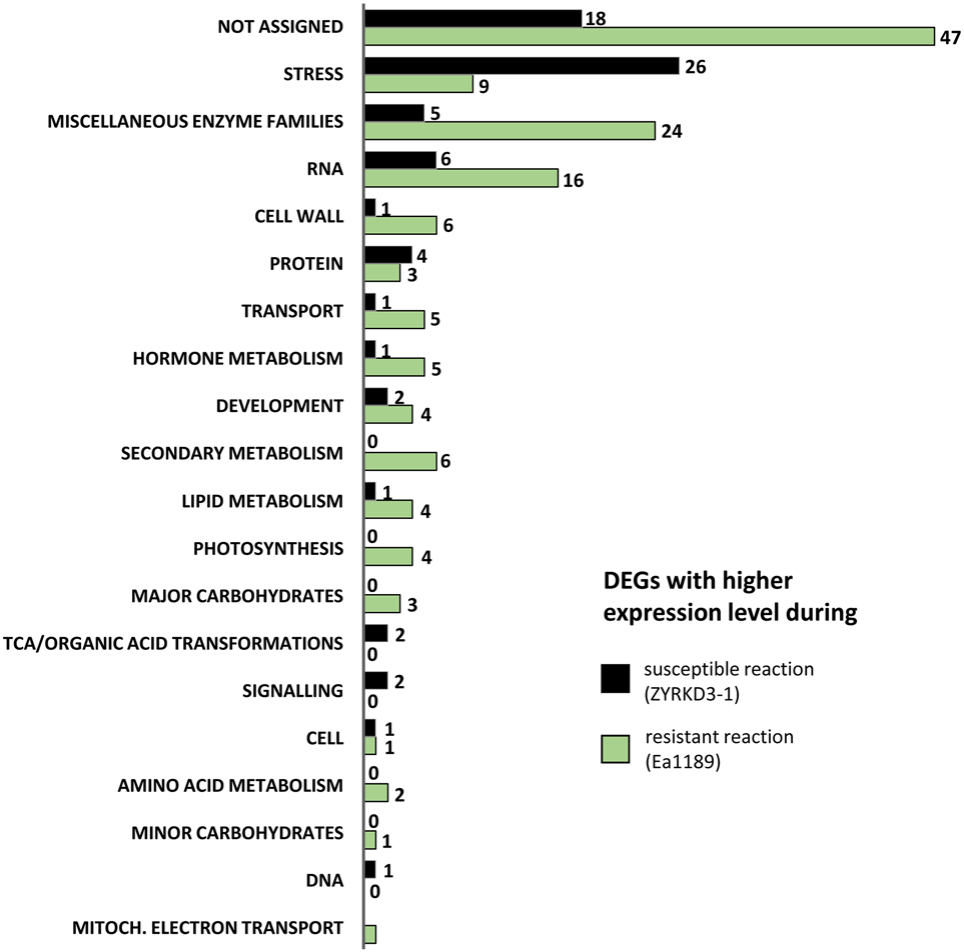
Functional categorization of differentially expressed genes in Mr5 during susceptible and resistant reaction to *Erwinia amylovora*. The functional categorization of genes (BIN) that were significant differentially expressed (DEGs) was performed by analysis with MapMan. The numbers of genes, which have a increased expression level during resistant reaction (after inoculation with Ea1189) or susceptible reaction (after inoculation with the *avrRpt2*_*EA*_ deletion mutant ZYRKD3-1), are depicted for each observed functional category. Figure 2 was created with Excel 2016 and PowerPoint 2016.

All identified DEGs were assigned to functional categories, so called BINs^18^ (Table S1). A large proportion of the DEGs (40%, 86 genes) are dedicated to the functional groups ‘stress’ (35 genes), ‘miscellaneous enzyme families (MISC)’ (29 genes) and ‘RNA’ (22 genes). Furthermore, for about 30% of all identified DEGs (65 genes), a functional assignment was not possible. The BINs ‘cell wall’, ‘protein’, ‘hormone metabolism’, ‘secondary metabolism’ and ‘transport’ represent groups with a moderate membership (each 6–7 genes). The residual DEGs are distributed to diverse functional groups as shown in Fig. 2.

The comparison of the proportion of genes with higher expression during the resistant reaction and the susceptible reaction displays a different distribution regarding the functional groups (Fig. 2; Table S1). During the susceptible reaction after inoculation of Mr5 with the virulent *avrRpt2*_*EA*_ deletion mutant ZYRKD3-1, DEGs which are assumed to be involved in stress response are overrepresented, particularly at 48 hpi (1 DEG at 2 hpi, 25 DEGs at 48 hpi; Table S1). Other functional categories seems to be more relevant during resistant response. DEGs with high expression levels associated with ‘MISC’, ‘RNA’, ‘cell wall’, ‘transport’, ‘hormone’ and ‘secondary metabolism’ are overrepresented during resistant reaction compared to susceptible reaction. Within the functional groups ‘RNA’, ‘transport’, ‘hormone’ and ‘secondary metabolism’, the majority of the genes are differentially expressed early after inoculation (2 hpi, Table S1). In Table S1, the categorization to the respective BINs is indicated for each identified DEG. In Table S2, DEGs are grouped to their functional BIN and furthermore, similarities to proteins from *Arabidopsis thaliana* and other plants are given.

### Heat-shock response is activated during susceptible reaction independent of AvrRpt2_EA_

Twenty six DEGs with increased expression during a susceptible reaction after inoculation with the *avrRpt2*_*EA*_-deleted mutant strain are categorized as stress response genes. Surprisingly, most of these genes (24 out of 26) are associated to the response against the abiotic stressor heat (Tables 2 and S2). A deeper view on the suggested similarities to proteins from *A. thaliana* reveal that most of these genes (21) are potentially coding for heat-shock proteins (HSPs) from diverse heat-shock protein families. Except of one, all DEGs were identified at 48 hpi (Table S1). A high degree of similarity to the *A. thaliana* counterparts could be observed for the differential expressed apple genes (MDP0000303430, MDP0000254260, MDP0000217508, MDP0000122734 and MDP0000265759) which are linked to *AtHSP90.1* (At5g52640), *AtHSP101* (At1g74310), *AtHSP70* (At3g12580) and *AtHSP70b* (At1g16030), respectively (Table 2). Consistent with this finding, four of the six DEGs with higher expression during susceptible response, namely MDP0000119199, MDP0000122783, MDP0000243895, MDP0000489886, MDP0000925901, are assigned to the functional group ‘RNA’, belong to the heat-shock transcription factor (HSF) family and show similarity to AtHSFA6B (At3g22830) or *AtHSFA2* (At2g26150), respectively. In addition, the gene MDP0000915991 shares similarity with *AtBAG6*, which is a chaperone regulator and known to be induced by heat. In contrast, during resistant response, none of the DEGs is associated with heat-shock proteins or heat-shock transcription factor family.

**Table 2:** DEGs categorized to functional groups ‘RNA’, ‘stress’ and ‘secondary metabolism’. All identified differential expressed genes categorized by MapMan to the BINs ‘RNA’, ‘stress’ and ‘secondary metabolism’ are displayed and filtered after their differential expression level, either during resistant reaction (after inoculation with wild type strain Ea1189) or susceptible reaction (after *avrRpt2*_*EA*_ deletion mutant strain ZYRKD3-1). The functional description of the sub-BIN and the degree of similarity to proteins from *A. thaliana* is given.

Gene	Functional description (sub-BIN)	Similarities to proteins from A. thaliana or other plant proteins
**DEGs with higher expression level during resistant reaction**		
BIN 'RNA' (16), all of them ‘regulation of transcription’		
MDP0000280307	Homeobox TF family	AT4G08150***, KNAT1 (KNOTTED-LIKE FROM ARABIDOPSIS THALIANA)
MDP0000254847	Homeobox TF family	AT1G23380**, KNAT6
MDP0000294096	Homeobox TF family	AT1G23380***, KNAT6
MDP0000737128	Homeobox TF family	AT5G15150**, HB-3 (HOMEOBOX 3)
MDP0000138651	Homeobox TF family	AT2G22430**, HB6 (HOMEOBOX 6)
MDP0000316497	Homeobox TF family	AT2G46680**, HB-7 (HOMEOBOX 7)
MDP0000272542	Homeobox TF family	AT2G27990***, BLH8 (BEL1-LIKE HOMEODOMAIN 8)
MDP0000136226	Homeobox TF family	AT1G62360***, STM (SHOOT MERISTEMLESS)
MDP0000204699	MYB domain TF family	AT5G15310***, ATMYB16 (MYB DOMAIN PROTEIN 16)
MDP0000716457	MYB domain TF family	AT5G15310***, ATMYB16 (MYB DOMAIN PROTEIN 16)
MDP0000944210	Basic Helix-Loop-Helix family	AT1G72210***
MDP0000160256	Basic Helix-Loop-Helix family	AT1G72210***
MDP0000212178	ARR	AT5G62920***, ARR6 (RESPONSE REGULATOR 6)
MDP0000136037	AS2, Lateral Organ Boundaries Gene Family	AT3G02550***, LBD41 (LOB DOMAIN-CONTAINING PROTEIN 41)
MDP0000307705	Chromatin assembly factor group	AT1G04880***
MDP0000827400	APETALA2/Ethylene-responsive element binding protein family	AP2, no original description
BIN 'stress' (9)		
MDP0000184034	Abiotic, unspecified	AT5G15780** (pollen Ole e 1 allergen and extensin family protein)
MDP0000219522	Abiotic, unspecified	AT5G15780** (pollen Ole e 1 allergen and extensin family protein)
MDP0000216647	Abiotic, unspecified	AT5G15780** (pollen Ole e 1 allergen and extensin family protein)
MDP0000165381	Abiotic, unspecified	AT2G34700** (pollen Ole e 1 allergen and extensin family protein)
MDP0000236390	Abiotic, unspecified	AT3G05950** (germin-like protein)
MDP0000937986	Abiotic, cold	AT5G52300*, LTI65 (LOW-TEMPERATURE-INDUCED 65)
MDP0000158507	Biotic	RICI_RICCO*** (Ricin precursor)
MDP0000711911	Biotic	RICI_RICCO*** (Ricin precursor)
MDP0000782642^1^	Biotic	AT5G38280***, PR5K
BIN 'secondary metabolism' (6)		
MDP0000440654	Flavonoids, dihydroflavonols	AT5G42800****, DFR (DIHYDROFLAVONOL 4-REDUCTASE)
MDP0000205617	Isoprenoids, terpenoids	AT5G23960***, TPS21 (TERPENE SYNTHASE 21)
MDP0000120176	Isoprenoids, terpenoids	AT5G23960***, TPS21 (TERPENE SYNTHASE 21)
MDP0000919962	Isoprenoids, terpenoids	AT5G23960**, TPS21 (TERPENE SYNTHASE 21)
MDP0000265187	Isoprenoids, terpenoids	AT4G15870**, ATTS1
MDP0000128578	Phenylpropanoids, lignin biosynthesis	AT1G67980**, CCoAMT (caffeoyl-CoA O-methyltransferase)
**DEGs with higher expression during susceptible reaction**		
BIN 'RNA' (6)		
MDP0000243895	Regulation of transcription, Heat-shock TF family	AT2G26150***, HSFA2
MDP0000489886	Regulation of transcription, Heat-shock TF family	AT2G26150***, HSFA2
MDP0000119199	Regulation of transcription, Heat-shock TF family	AT3G22830***, HSFA6B
MDP0000925901	Regulation of transcription, Heat-shock TF family	AT3G22830**, HSFA6B
MDP0000217497	Processing splicing	AT1G80070*****, SUS2 (ABNORMAL SUSPENSOR 2)
MDP0000122783	RNA binding	AT5G55550** (RNA recognition motif-containing protein)
BIN 'stress' (26)		
MDP0000303430	Abiotic, heat	AT5G52640*****, HSP90.1 (heat shock protein 90.1)
MDP0000254260	Abiotic, heat	AT5G52640*****, HSP90.1 (heat shock protein 90.1)
MDP0000217508	Abiotic, heat	AT1G74310*****, HSP101 (heat shock protein 101)
MDP0000122734	Abiotic, heat	AT3G12580****, HSP70 (heat shock protein 70)
MDP0000265759	Abiotic, heat	AT1G16030****, HSP70b (heat shock protein 70B)
MDP0000290546	Abiotic, heat	AT2G20560*** (DNAJ heat shock family protein)
MDP0000795157	Abiotic, heat	AT2G20560***, (DNAJ heat shock family protein)
MDP0000149486	Abiotic, heat	AT4G27670**, HSP21 (heat shock protein 21)
MDP0000214382	Abiotic, heat	AT4G27670**, HSP21 (heat shock protein 21)
MDP0000166796	Abiotic, heat	AT2G29500** (17.6 kDa class I small heat shock protein)
MDP0000207407	Abiotic, heat	AT2G29500** (17.6 kDa class I small heat shock protein)
MDP0000158520	Abiotic, heat	AT5G59720**, HSP18.2 (heat shock protein 18.2)
MDP0000265157	Abiotic, heat	AT5G59720**, HSP18.2 (heat shock protein 18.2)
MDP0000791550	Abiotic, heat	AT5G59720**, HSP18.2 (heat shock protein 18.2)
MDP0000810697	Abiotic, heat	AT5G59720**, HSP18.2 (heat shock protein 18.2)
MDP0000323296	Abiotic, heat	AT5G59720**, HSP18.2 (heat shock protein 18.2)
MDP0000604702	Abiotic, heat	AT5G12020**, HSP17.6II (17.6 kDa class II heat shock protein)
MDP0000362505	Abiotic, heat	AT5G12020**, HSP17.6II (17.6 kDa class II heat shock protein)
MDP0000700383	Abiotic, heat	AT5G12020**, HSP17.6II (17.6 kDa class II heat shock protein)
MDP0000125300	Abiotic, heat	AT4G25200**, HSP23.6-MITO (mitochondrion-localized small heat shock protein 23.6)
MDP0000291831	Abiotic, heat	AT5G12030**, HSP17.6A (heat shock protein 17.6A)
MDP0000278972	Abiotic, heat	AT1G56410*, ERD2 (EARLY-RESPONSIVE TO DEHYDRATION 2)
MDP0000549793	Abiotic, heat	AT3G08970***, ATERDJ3A
MDP0000915991	Abiotic, heat	AT2G46240**, BAG6 (BCL-2-ASSOCIATED ATHANOGENE 6)
MDP0000644109	Abiotic	AT5G20150***, SPX1 (SPX DOMAIN GENE 1)
MDP0000521048	Abiotic, unspecified	AT5G20630***, GER3 (GERMIN 3)

(*) very weakly similar, (**) weakly similar, (***) moderately similar, (****) highly similar, (*****) nearly identical to protein from *Arabidopsis thaliana*; TF (transcription factor); ^1^ moderately similar to Thaumatin-like protein 1a precursor (Allergen Mal d 2) from *M. domestica.*

### Presence of different hormone pathways during susceptible and resistant reaction

One differential expressed gene MDP0000277666 is grouped to the BIN ‘hormone metabolism’ and was higher expressed during the susceptible reaction (Table S2). This gene is highly similar to the *AtLOX2* (At3g45140, LIPOXYGENASE 2) and therefore might related to jasmonate metabolism. In contrast, five DEGs probably related to hormone metabolism showed increased gene expression levels during resistant response after. These are associated with different hormones such as gibberilin, auxin and ethylene, but not with jasmonate and salicylic acid (Table S2).

### Role of genes particularly active during resistant reaction

As described before, a large number of DEGs are grouped in the BIN ‘MISC’, whereas the number of DEGs during resistant reaction is much higher as compared to the susceptible reaction. The predicted functions of the DEGs grouped into the category MISC are versatile (Table S2), including GDLS-motif lipase genes (6 DEGs), phosphatase genes (3 DEGs), and UDP-glucosyl and -glucoronyl transferase genes (3 DEGs), the latter showed high expression level during both-, resistant and susceptible reaction. The prevalence of cytochrome P450 genes (6 DEGs) within the MISC group had increased expression levels only during resistant reaction.

Furthermore, DEGs categorized within the BIN ‘secondary metabolism’ are only identified during resistant reaction and may be associated with terpenoids (4 DEGs), lignin biosynthesis (1 DEG) and flavonoids (1 DEG) (Table 2, Table S2). The gene related to flavonoids, MDP0000440654, shares high similarity to the dihydroflavonol 4-reductase *AtDFR* (At5g42800). Three DEGs are homologous to the terpene synthase 21 from *A. thaliana* (*AtTPS21*, At5g23960).

Compared to susceptible response, more transcription factors (BIN ‘RNA’) with increased expression level were identified during resistance response (12 genes), such as the Homeobox transcription factor family (8 genes), the MYB domain transcription factor family (2 genes) and the Basis-Helix-Loop-Helix family (2 genes) (Table 2). Interestingly, some identified apple genes categorized to the stress response during resistant reaction are mapped to different genes linked to allergens including four genes coding for pollen Ole e 1 allergene and extensin family proteins (Table 2). Furthermore, a gene MDP0000782642 similar to *PR5K* from *A. thaliana* (Table 2) is coding for thaumatin-like protein. Additional alignments of this protein display a high identity of 77% amino acids to the homologous protein MdTL1, which was identified as a Mal d 2 allergen^19^.

### Evaluation of regulated genes in response to *E. amylovora*

A subset of DEGs with increased expression during resistant response was further analyzed by a high-throughput real-time qPCR. Primers were designed for 106 DEGs, tested and verified by RT-PCR and qPCR. Finally, 81 primer pairs could be established for gene expression analysis. To analyze the resistant response, Mr5 plants were inoculated with the avirulent wild type strain Ea1189 and expression of the genes was compared to the not-inoculated control at 1, 2, 4, 12, 24 and 48 hpi.

**Figure 3:**
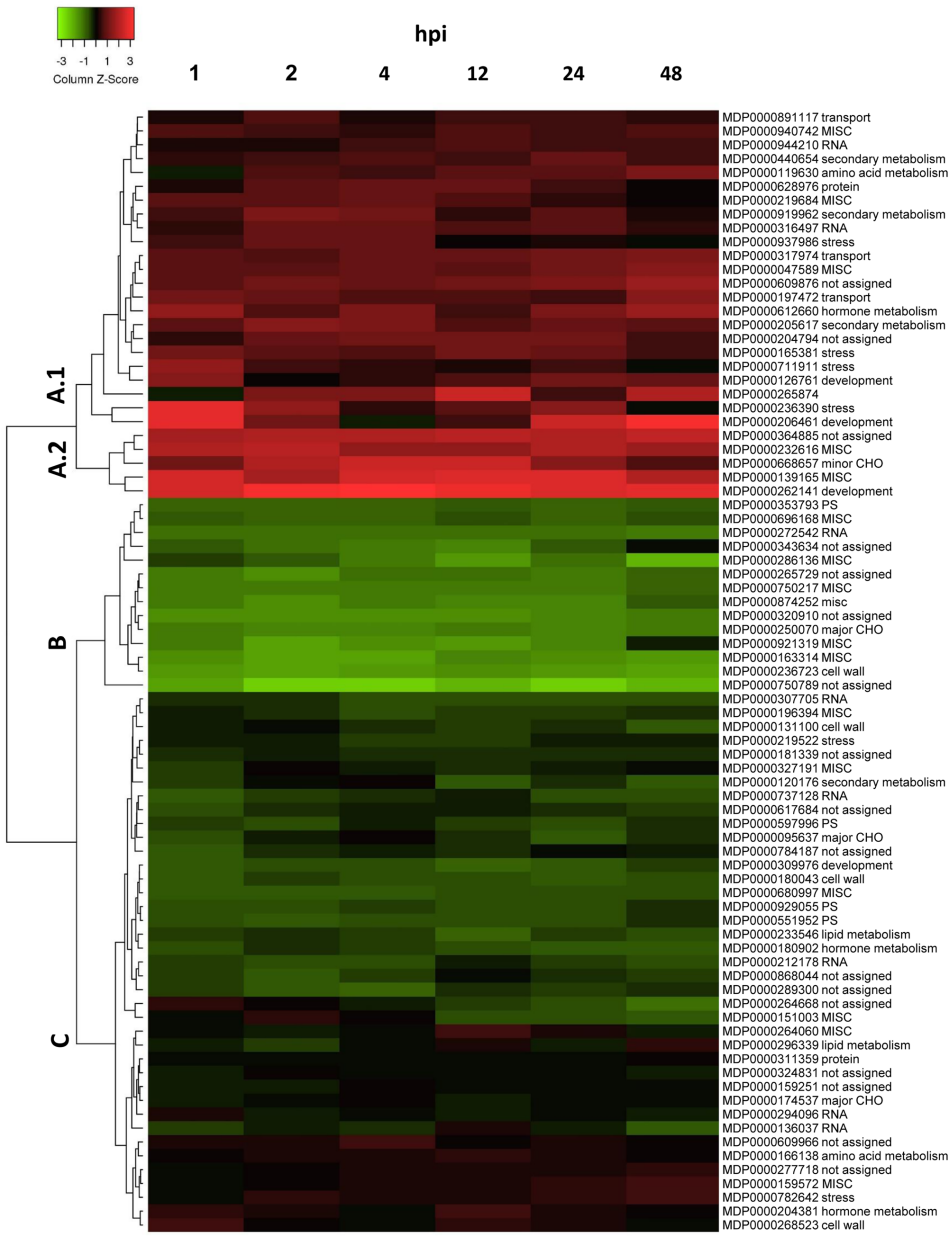
Change of expression of DEGs during resistant reaction. Mr5 plants were inoculated with the avirulent Ea1189 wild type strain and the expression of selected genes was determined by high-throughput real-time qPCR at 1, 2, 4, 12, 24 and 48 hpi. The heat map represents the mean log2 fold change compared to the non-inoculated control. The cluster A contains 28 genes that exhibited induced expression after inoculation as compared to the non-inoculated control whereas 14 genes of cluster B showed a reduced expression. The expression of genes clustered in C was similar to the non-inoculated control. Figure 3 was created using Heatmapper Tool (http://www.heatmapper.ca/expression/) and PowerPoint 2016.

**Figure 4:**
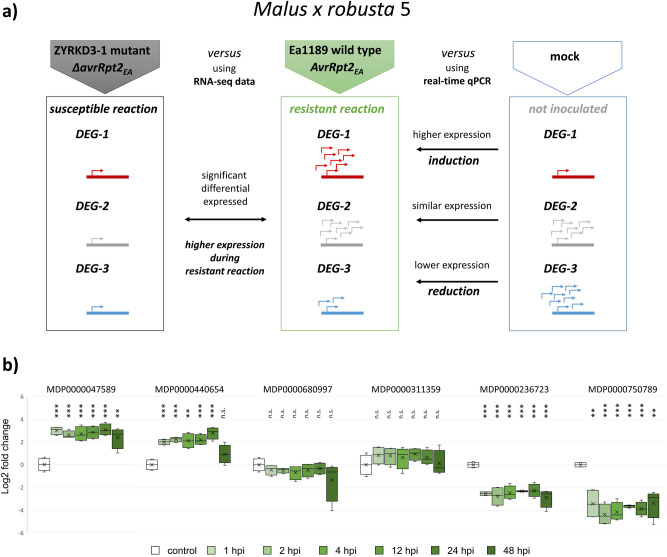
Schematic examples of expression patterns of DEGs with high expression levels during resistant reaction obtained by RNA-seq compared to gene expression determined by qPCR. (**a**) Genome-wide gene expression was measured by RNA-seq to identify genes which are differentially expressed during resistant reaction compared to susceptible reaction. The comparison of expression level between non-inoculated and inoculated condition (avirulent wild type strain Ea1189) reveal the impact on modulation of gene expression (induction, reduction) during resistant response. (**b**) Gene expression of a set of DEGs with an increased expression during resistant reaction was determined by qPCR at 1, 2, 4, 12, 24 and 48 hpi and the fold change was calculated relatively to the non-inoculated control. The log2 fold change is depicted in the box plot diagram and significant differences to the control were tested by t-test (*p*-values < 0.01 are marked with **, < 0.001 with ***, ≥ 0.05 with n.s for not significant). Figure 4 was created with Excel 2016 and PowerPoint 2016.

The heatmap (Fig. 3) shows an overview of the change of gene expression by inoculation of Mr5 with the avirulent strain Ea1189 for each gene. Genes were clustered according to their similarities in expression pattern. Three main clusters were characterized by genes with an induced (cluster A, 28 genes), a reduced (cluster B, 14 genes) and a similar (cluster C, 39 genes) gene expression as compared to the not-inoculated control, indicating the differences between RNA-seq data and qPCR data (Fig. 4).

Regarding a potential role in the resistance mechanism against the pathogen, a special interest is on genes in cluster A, showing increased expression after inoculation (Fig. 3). The magnitude of change in expression as well as the time point of induction in gene expression differed in cluster A. Cluster A can be divided in two sub-clusters A.1 and A.2. Sub-cluster A.1 includes genes with a moderate induction (temporary or general) as well as genes with a temporary strong induction. Interestingly, three genes probably coding for enzymes involved in secondary metabolism linked to dihydroflavonols (MDP0000440654) and terpenoids (MDP0000205617, MDP0000919962) are grouped within this cluster and showed a general moderate induction after inoculation. The genes which exhibit a temporary induction after inoculation are e.g. MDP0000711911 (type 2 ribosome-inactivating protein Md2RIP^20^, MDP0000265874 (apple dehydrin MdDHN6^21^), MDP0000236390 (coding for a germin-like protein) and MDP0000206461 (coding for a bidirectional sugar transporter). The five genes of cluster A.2 exhibited a general strong induction after infection. The function of MDP0000364885 is not assigned and additional BLAST searches did not lead to significant hits whereas the other genes of these group were assigned as GDLS-motif lipase gene (MDP0000232616), inositol oxygenase 1-like gene (MDP0000668657), plant lipid transfer protein/hydrophobic protein helical domain (MDP0000139165) and SQUAMOSA promoter binding protein MdSBP6 (MDP0000262141^22^).

## Discussion

Plants health is a major topic with the environment, end hunger, reduce poverty and boost economic development. The pome fruit apple is one of the most important fruit crops worldwide with a yield of 85 million tons per year (Food and Agriculture Organization, FAO). Most of the commercial apple cultivars are highly susceptible to fire blight, which is present in more than 50 countries^23^ and regarded as the most devastating bacterial disease affecting pome fruits with economic losses, like in Switzerland where an outbreak in 2007 resulted in costs of about US$27.5 million^3^. Disease control strategies include the application of antibiotics such as streptomycin, kasugamycin or oxytetracycline, which are permitted for conventional apple production in the US^24^, but banned or strictly regulated in most European countries. The most sustainable and environmentally friendly alternative is breeding and subsequent cultivation of fire blight resistant apple cultivars^2^. To this aim, the present study will contribute to the understanding of the molecular basics of the resistant reaction of the plant attacked by the pathogen, which will help to develop future strategies for resistance breeding.

The *Malus*-*E. amylovora* host–pathogen relationship is a complex system and differential interactions among *Malus* genotypes and *E. amylovora* strains have been reported^5,25^. Furthermore, distinct types of fire blight resistance mechanisms were found in *Malus* as well as the *E. amylovora* strains differ in virulence^5,25^. The host–pathogen system of the fire blight resistant wild apple genotype Mr5 to *E. amylovora* is the most studied^6,13–15,25–27^ and therefore, represents a good model system to discover fire blight resistance response of the plant. Moreover, the bacterial effector AvrRpt2_EA_ was identified as determining factor for the induction of fire blight resistance of Mr5^13^. In this work, the presence and absence of *AvrRpt2*_*EA*_ in the bacterial strain was used as molecular switch to specifically study the reactome in the same apple genotype during resistant and susceptible reaction, respectively. The comparison of both transcriptomic profiles, which were obtained by RNA-seq at different time points after inoculation of Mr5 resulted in the identification of 211 DEGs (Table S1). Most of them were found to exhibit increased expression during resistance reaction in comparison to susceptible reaction (Table S2). As the mapping of the RNA-seq reads to annotated apple genes were performed using the reference transcriptome of 'Golden Delicious'^17^, therefore it is possible that genes only present in Mr5 genotype were not detected as DEGs. In contrast to our study, Silva et al.^28^ analyzed the difference in response of two apple cultivars inoculated with one highly virulent *E. amylovora* strain.

In this study, a series of genes associated with heat-shock response and similar to the *A. thaliana* homologs *HSP90.1*, *HSP101*, *HSP70*, *HSP70b*, *HSFA6B* and *HSFA2* were found to be differentially expressed after infection with *E. amylovora* (Table 2) and exhibted increased expression during susceptible reaction. HSPs were originally identified as proteins strongly increased by heat. Meanwhile they are known to be also induced in response to almost all kind of stresses, including abiotic and biotic of stresses^29^. HSPs are characterized as molecular chaperones avoiding misfolding of other proteins. An involvement of HSPs in plant defense response and disease resistance was described by various studies (for review see^30^). For HSP90, an important role in modulating the structure and/or the stability of R proteins was suggested^31^. In *Arabidopsis thaliana*, HSP90.1 was described to be required for disease resistance mediated by the R-protein RPS2^32^, which results in hypersensitive response mediated cell death. Interestingly, Gardiner, et al.^33^ identified members of the HSP90 gene-family associated with the fire blight resistance QTLs of Mr5 on LG3 and LG7. The single nucleotide polymorphism (SNP) marker NZsnEB151679 could be identified co-localizing with MDP0000303430, a *HSP90* gene-family member on LG7^34^ and this gene was also identified in the present study as a DEG. HSP90 is part of a protein complex, which was found to be necessary for the regulation of some disease resistance NB-LRR proteins in plants^34,35^. Further investigation of the role of HSP90 in susceptibility and/or resistance to fire blight is of high interest. Therefore, treatments with geldanamycin, which is a specific inhibitor of the HSP90 ATPase activity^36^, may help to evaluate the loss of HSP90 function in apple. A function in plant defense response, which is distinct from HSP90, was also shown for HSP70^29^.

The expression of HSPs is primarily regulated by specific heat shock factors (HSFs) that bind to heat stress elements (HSEs) located in the promotor of HSPs and HSFs^37^. According to the finding that a number of HSPs are differentially expressed, also HSFs were found to be differentially expressed in this study. An involvement of HSFs was suggested in a pathway that is associated with controlled cell death triggered by pathogen infection and they may act as sensor of reactive oxygen species^38^. A suppression of plant death caused by the pathogen was described by deactivation of the heat shock protein factor HSFA2^39^.

A gene similar to *AtLOX2* was found to be higher expressed during susceptible reaction independent of AvrRpt2_EA_. Also Kamber et al.^40^ identified a differently expressed gene in the susceptible apple cultivar 'Golden Delicious' in response to *E. amylovora* that shares similarity to *AtLOX2*. This gene potentially codes for a chloroplastic lipoxygenase 2, which is required for the biosynthesis of jasmonic acid^41^. It was shown that *AtLOX2* is highly expressed during susceptible reactions to pathogens^42^.

Among the 140 DEGs that show increased expression during resistance response in comparison to the susceptible reaction, the expression of 81 genes were further investigated by high-throughput real-time qPCR. Differentially regulated genes, which were induced specifically during resistance response compared to the not-inoculated control may of high interest (Fig. 3, in total 28 genes of cluster A).

One of these genes codes for a DFR enzyme, which was described to be involved in the formation of flavonoids and supposed to be responsible for enhanced resistance against fire blight^43^. DFR is one of the rate-limiting enzymes catalyzing the reduction of dihydroflavonols to flavan-3,4-diols and plays a key role in the formation of common and condensed anthocyanins^44^. Furthermore, an induction of the *DFR* gene expression could be observed after inoculation with the avirulent *E. amylovora* strain (Fig. 3; Fig. 4B). These results indicate that expression of *DFR* (MDP0000440654) was induced in response to the pathogen, potentially leading to increased biosynthesis of anthocyanins. A general role of anthocyanins in plant disease resistance was described before^45^. In *Malus* wild species, an accumulation of anthocyanins were correlated with rust resistance^46^. Accumulation of flavonoids such as flavone, flavonol, flavanols, procyanidins and anthocyanins is regulated by members of the MYB and basic helix-loop-helix (bHLH) transcription factor families^47^. Consistently, RNA-seq data revealed that two DEGs each coding for MYB16 and for bHLH transcription factors showed increased expression during resistance response (Table 2), suggesting an involvement in fire blight disease resistance response, potentially by regulating flavonoid biosynthesis. For one of them (MDP0000944210, bHLH), an induction of gene expression after inoculation with *E. amylovora* was confirmed by qPCR (Fig. 3).

Another class of DEGs coding for enzymes connected to secondary metabolism were identified exhibiting high expression levels specifically during resistant response, namely terpene synthase 1 (ATTS1) and 21 (TPS21, three different DEGs). For two *TPS21* genes (MDP0000205617, MDP0000919962), an induction of gene expression after the inoculation with the avirulent *E. amylovora* strain were confirmed by qPCR (Fig. 3). The functional spectrum of terpenoids and involved metabolic enzymes is huge. Nevertheless, TPS21 is known to encode for a sesquiterpene synthase producing the volatile terpene (*E*)-ß-caryophyllene, which was shown to have a defense activity against diverse pathogens^48^. The ectopic expression of *TPS21* in *A. thaliana* leads to enhanced emission of (E)-ß-caryophyllene. Furthermore, it was shown to inhibit the growth of *Pseudomonas syringae* and enhances resistance against bacterial pathogen^49^. In addition, formation of (E)-ß-caryophyllene in apple flowers was shown after honeybee-mediated dispersal of *Erwinia amylovora*. Altogether, it seems likely that induction of *TPS21* gene expression leads to the formation of (E)-ß-caryophyllene, promoting disease resistance by its antimicrobial activity. Further investigations on the role of the identified genes and their regulation may be of high interest for fire blight resistance breeding.

Six genes of cytochrome P450 (namely MDP0000151003, MDP0000286136, MDP0000563385, MDP0000661381, MDP0000750217, MDP0000874252, Table S2) were differentially expressed during resistant response. These genes belong to the largest gene families in plants, represented by 244 genes in *A. thaliana*, and they are coding for membrane bound monooxygenases involved in numerous biosynthetic and xenobiotic pathways^50^. Because of the functional diversity of these enzymes, it is difficult to define a specific function, but the involvement of P450 proteins in resistance to pathogens seems likely. A series of P450 enzymes are involved in terpenoid metabolism^51^. Two DEGs encoding for ricin precursors (Table 2) belong to the family of ribosome-inactivating enzymes (RIPs) and exhibited increased expression during resistance response in the RNA-seq analysis. For one of them, an induction of gene expression by the avirulent strain was demonstrated by qPCR (Fig. 4). RIPs are widely spread in the plant kingdom and type 1 and type 2 RIPs are present in *Rosaceae*^21^. They are toxic N-glycosidases that depurinate eukaryotic and prokaryotic rRNAs, leading to the arrest of protein synthesis and play a significant role in defense against bacterial pathogens^52^. The identified DEG MDP0000711911, belongs to type 2 of RIPs and is described as Md2RIP^52^. A biological activity on ribosomes was demonstrated for Md2RIP as the protein synthesis was inhibited in the presence of Md2RIP^21^. In a different study, it was shown that heterologous expression of *RIPs* from apple led to enhanced resistance of tobacco plants to the armyworm *Spodoptera exigua* and that these apple RIPs exerted high larval mortality^53^. To our knowledge, data from this work demonstrated an induction of *RIP* gene expression in apple by a pathogen and suggested further investigations on the role of RIPs in resistance of apple to *E. amylovora* and other pathogens. As apple RIPs may also have a toxic effect to human cells, an additional focus should be on the effect of resistance breeding to human health.

As the data of this study revealed, apple resistance response triggered by effector AvrRpt2_EA_ is versatile on transcriptomic level. Most likely, the recognition of AvrRpt2_EA_ by a R-protein induces resistance signaling framework and initiates immune response, defense relay and amplification of the signal^9^. However, potential constitutive expressed genes important for resistance response, as it is the case for many R-proteins, may not be detected by this approach. Nevertheless, it is obvious that transcription factors may play an essential role in orchestrating diverse resistance reactions. In this context, gene MDP0000262141 coding for a squamosa promoter binding like (SPL) transcription factor is of interest. This differentially expressed gene exhibited a high expression level specifically during resistant reaction and was the most induced gene after inoculation with the avirulent strain. It is known from other plant species, that SPL transcription factors are involved in resistance response and positively modulate defense gene expression^9,54^. It was proposed that a cytoplasmatic NLR receptor is activated after effector recognition, and then relocates from the cytoplasm to the nucleus where it interacts with the SPL transcription factor leading to the activation of defense gene expression. A function of MDP0000262141 in such an early step of resistance response needs to be investigated.

In summary, a row of apple genes were identified within this work, which might be important for the susceptible as well as the resistant response to the pathogen. Furthermore, this work poses some candidates including genes coding for enzymes involved in formation of flavonoids and terpenoids, RIPs and a SPL transcription factor that may be crucial for the resistance of the apple plant challenged by *E. amylovora*. Future studies may elucidate their potential role in the fire blight resistance mechanism of Mr5.

## Materials and methods

### Plant material

Shoots of Mr5 (MAL0991) were grafted onto certified M9 rootstocks, which were obtained from a nursery. Plants were transferred in the greenhouse in Quedlinburg and grown at temperatures between 10 and 15 °C under a natural photoperiod with extension of daytime in spring.

### Strains and inoculation

*E. amylovora* wild type strain Ea1189 and the *avrRpt2*_*EA*_ mutant strain ZYRKD3-1^11^ were used for fire blight inoculations. Bacteria were cultivated on bouillon glycerin agar at 28 °C for 48 h. For the mutant strain ZYRKD3-1, 20 µg/ml chloramphenicol was added to the growing media. Actively growing shoots with a minimum length of 25 cm were inoculated by cutting off the tips of two youngest leaves with scissors immersed in the bacterial suspension (10^9^ cfu/ml). Plants were maintained in the greenhouse at 27 °C (day) and 22 °C (night).

### Sample preparation and RNA extraction

Two inoculated leaves were collected and pooled at 1, 2, 4, 12, 24 and 48 h post inoculation (hpi) from each ten plants per time point, per inoculation and per biological replicate. Additionally, leaves were collected from each ten shoots of Mr5 without inoculation per biological replicate. Samples were immediately frozen in liquid nitrogen. The frozen plant material was homogenized in a 15 ml Falcon tube by grinding with a glass rod. An amount of around 100 μg of the homogenized material was used for RNA isolation with the InviTrap Spin Plant RNA Mini Kit (Stratec Molecular GmbH). RNA was treated with DNA-free Kit (Life Technologies GmbH) to remove remaining DNA. The quality of the RNA was verified with the Bioanalyzer 2100 (Agilent Technologies) and revealed in a RNA Integrity Number (RIN) of all samples > 8.0.

### cDNA library construction and RNA sequencing

The transcriptome of Mr5 inoculated with the wild type strain Ea1189 and the avrRpt2_EA_ mutant strain ZYRKD3-1 was determined at 2 and 48 hpi. TruSeq RNA Sample Preparation Kit (Illumina) was used for construction of the NGS library from a pool of ten plants each per inoculation and time point following the manufacturer’s instruction. The barcoded libraries were pooled and sequenced on one paired-end lane with a read length of 50 bp using the Illumina HiSeq2000 system. Reads passing standard filtering of the sequencer were cleaned for adapter sequences by trimming and subjected to successive bioinformatics analysis. Library construction and sequencing was done by GATC Biotech AG.

### RNA-seq data analysis and bioinformatics

Quality of the raw RNA-seq reads was assessed using FastQC 0.10.1. The paired-end reads were mapped to the reference transcriptome of *M. domestica* cv. 'Golden Delicious' (Malus_x_domestica.v1.0.consensus_CDS.fa.gz) by Burrows-Wheeler Alignment tool (BWA) with the default parameters^55^. Only uniquely aligned reads were utilized for differential gene expression analysis using DESeq R package [vers. 3.0.2]^56^. The variance was estimated by assuming no replicates. The samples inoculated with the wild type strain and the mutant strain were compared at 2 and 48 hpi. To test for differential expression, count data were fitted to the negative binomial distribution. P-values for the statistical significance of the fold change were adjusted for multiple testing with the Benjamini–Hochberg correction for controlling the false discovery rate of < 10%^57^. The assignment of differentially expressed genes to pathways was performed with MapMan^19^. The annotation to homologue genes of *A. thaliana* by Phytozome v9.0^57^ of *M. domestica* was used for the assignment (Mdomestica_196-2.txt).

### Primer development and optimization for qPCR

Primer pairs were designed using Primer3. The main parameters were determined as followed: primer length 18–25 bp (optimum 20); GC content 40–60%, product size 100–200 bp (optimum 150 bp), melting temperature 60 ± 1 °C (temperature difference < 0.5 °C). The self-complementarity score was set to three with an increased value if no acceptable primers were found. Primer pairs were also tested by NetPrimer (PREMIER Biosoft International, Palo Alto, CA) to avoid hairpins, primer dimers and primer cross dimers. The 106 primer pairs were designed on the transcriptome of 'Golden Delicous' and corrected in the case of differences to the transcriptome of Mr5. Primer verification was performed firstly by Reverse Transcriptase-PCR (RT-PCR) (94 °C for 3 min/30 s, 57 °C for 1 min, 72 °C for 1.30/5 min, 30/35 cycles) with one biological replicate of each sample. Positively tested primer pairs were further analyzed by quantitative real-time qPCR (94 °C for 3/1 min, 61 °C for 1 min, 72 °C and 1 for, 40 cycles). To use primers in the BioMark HD System, the Ct value should not be higher than 35. The sequences of all primers used in this analysis are listed in Table S3.

### Gene expression analysis with BioMark HD system

Eighty-one DEGs identified in the transcriptome analysis were further validated by qPCR with the BioMark HD System (96.96 Dynamic Array Integrated Fluidic Chip; Fluidigm). Ubiquitin, GAPDH, EF1α, Rubisco, RNA-Polymerase (two different primer pairs) were used as reference genes. Two biological and two technical replicates of each sample as well as negative controls (cDNA synthesis without reverse transcriptase) and water were randomly spread on the chip.

For all targets, the forward and reverse primers were mixed for the pre-amplification step to a final concentration of 20 µM with low TE buffer (10 mM Tris, 0.1 mM EDTA, pH 8.0). Each primer pair (10 µl) was combined and filled up with low TE to 1 ml (200 nM pooled primer mix). 4.5 µl Pre-Mix, which consists of 2.5 µl TaqMan PreAmp Master Mix and 1.25 µl pooled primer mix and 1.5 µl cDNA were pre-amplified using the GeneAmp PCR 9700 System (ABI). The cycling conditions were set to 95 °C for 10 min, followed by 14 cycles with 95 °C for 15 s and 60 °C for 4 min. Afterwards, the reactions were diluted 1:20 with low TE buffer.

Preparation of the 96.96 Dynamic Array Integrated Fluidic Chip was performed according to the manufacturer’s instructions and loaded with the primer assay (6 µl) and the sample assay (6 µl). For the primer assay, 3.85 µl of the master mix (3.5 µl 2 × Assay loading reagent, 0.35 µl low TE) were complemented with 3.15 µl 20 µM primer mix. For the sample assay, 5.2 µl sample pre-mix solution (3.5 µl 2X TaqMan Gene Expression Master Mix (ABI), 20X DNA Binding Dye Sample Loading Reagent (Fluidigm), 20X EvaGreen DNA binding dye (Biotium) and 1X low TE) was mixed with 1,8 µl pre-amplified cDNA (diluted 1:20). The reaction was performed as followed: 50 °C for 2 min and 95 °C for 10 min, followed by 40 cycles of 95 °C for 15 s and 60 °C for 60 s. After amplification melt curve analysis was performed by heating the samples 1 °C per second from 60 to 95 °C. Data were analyzed by the Fluidigm Real-Time PCR Analysis 3.1.3 software (linear baseline correction, auto Ct threshold determination and quality threshold of 0.65). The specificity of PCR reactions was validated by analysis of melt curves and non-specific PCR reactions were excluded. The stability of the six included primer pairs for reference genes was analyzed using RefFinder, a tool that integrates the major computational programs geNorm, Normfinder, BestKeeper and the comparative Delta-Ct method^59^. This analysis leads to the selection of four reference genes (RNA-Polymerase, GAPDH, EF1α, Ubiquitin) for normalization of gene expression, with ranking values from 1.4 to 2.8 in the comprehensive ranking. Heatmap was generated using the Heatmapper Tool^60^ with the parameters scale type ‘column’, clustering method ‘complete linkage’ and distance measurement method ‘euclidean’.

### Ethical statements

The authors declare that the use of plants parts in the present study complies with international, national and/or institutional guidelines. All plant material used were gained from the orchard of the Julius Kühn Institute (JKI) – Federal Research Centre for Cultivated Plants, Institute for Breeding Research on Fruit Crops, except the rootstocks, which were delivered by a rootstock nursery.

## Supplementary Information

Supplementary Tables.

Supplementary Table S3.
