# Supplementary material for: Transcriptional profile of AvrRpt2EA-mediated resistance and susceptibility response to Erwinia amylovora in apple
Source: Sci Rep. 2021 Apr 22;11:8685. doi: 10.1038/s41598-021-88032-x (PMC8062453; doi:10.1038/s41598-021-88032-x)
Supplement: Supplementary file 2 — Supplementary Table S3. [file 41598_2021_88032_MOESM2_ESM.pdf]

Transcriptional profile of *AvrRpt2$_{EA}$*-mediated resistance and susceptibility response to *Erwinia amylovora* in apple

Susan Schröpfer[1,*], Isabelle Vogt[1,*], Giovanni Broggini[2], Andreas Dahl[3], Klaus Richter[4], Magda-Viola Hanke[1], Henryk Flachowsky[1] & Andreas Peil[1,✉]

[1]Julius Kühn Institute (JKI) – Federal Research Centre for Cultivated Plants, Institute for Breeding Research on Fruit Crops, Pillnitzer Platz 3a, 01326 Dresden, Germany

[2]ETH Zurich, Plant Pathology, Integrative Biology Zurich (IBZ), Universitätstrasse 2, 8092 Zurich, Switzerland

[3]DRESDEN-concept Genome Center, Center for Molecular and Cellular Bioengineering (CMCB), Technische Universität Dresden, Fetscherstr. 105, 01307 Dresden, Germany

[4]Julius Kühn Institute (JKI) – Federal Research Centre for Cultivated Plants, Institute for Resistance Research and Stress Tolerance, Erwin-Baur-Strasse 27, 06484 Quedlinburg, Germany

[*]these authors contributed equally to this work

[✉]Corresponding author: andreas.peil@julius-kuehn.de

Supplementary Information

Table S3: Primer sequences used in gene expression analysis with the BioMark HD system. Forward (F) and reverse (R) primer sequences as well as the size of the resulting PCR product [bp] are given for each analyzed gene.

Gene		sequences 5'-->3'			[bp]
Ubiquitin	F:	ATCACCCTAGAGGTCGAGTCTTCC	R:	GCGGAATGCCTTCCTTGTCTTG	76
GAPDH	F:	GGTTGCTAGGGTCGCTCTTCAG	R:	GTCCGTGGTGATGAAGGGATCG	73
EF1α	F:	TCAAGCGTGGGTACGTTGCTTC	R:	GATGACCTGAGCGATAAAGTTGGC	77
Rubisco	F:	TCCCTGTCACCAGAAAGAGCAAC	R:	AGGCCACACCTGCATACATTGC	80
RNA-Polymerase	F:	AGTTTGCTGAAGCTCCGTGTGC	R:	CGGAAGCGATTTGGAGGGATCAAG	76
RNA-Polymerase	F:	ATATGCCACCCCGTTCTCTACT	R:	CACGTTCCATTTGTCCAAACTT	286
MDP0000047589	F:	TCGGTGGAGATTCRGCTA	R:	GCAGGCTCAWACCARTGYGAC	151
MDP0000095637	F:	TGGAGACGCAGATARAACARC	R:	GAATGAGRCCGCAGGGTTC	147
MDP0000119630	F:	TTCGTTTGTGCTGCTGGATA	R:	CAACCCTTTCAGTTCCGATG	130
MDP0000120176	F:	AGAAGGCAAGTGAACAAAGCA	R:	TAGCCCACCCTyAGCAATAG	159
MDP0000126761	F:	CAACRCCAAGCTAGTCTGC	R:	TCTGGGTCKGTCATCACCAA	123
MDP0000131100	F:	RGGTGGTGAGGATGGAGATMGA	R:	ATCAGCRGGAAARAAATGG	142
MDP0000136037	F:	GAYAGCAAGGAATCGGAGAG	R:	AACCCAAGCGTCAGCTCYAA	140
MDP0000139165	F:	CCCACCAGATGGAGCCTAT	R:	AAGTTGCCACATACCCCTAGC	141
MDP0000151003	F:	ATCCCTGCGAAAACACARGT	R:	CAAACTCCTGACCYACGAAACC	132
MDP0000159251	F:	YGAGAAGGTGAAGGGGTTTG	R:	GGCGTGAAAGMACYGGCTTA	155
MDP0000159572	F:	AGCAACCTAACCGACGCTAA	R:	GGTATTGAAGGGGACGCATA	147
MDP0000163314	F:	SACCACCATCCYCCTCAAG	R:	CCTGACSCTACAGWCACAGAAA	178
MDP0000165381	F:	AGGAGCTTTGTAGCGGTTCA	R:	TGTGGTTTCCTTCACAACCA	153
MDP0000166138	F:	CRTCCTGGTCWCCGAAAAAC	R:	ACCACCTTCAACCTCCCTTT	195
MDP0000174537	F:	CCaAAACAYCACAAGCAGTC	R:	TATCAACAGGACCCCCATTT	169
MDP0000180043	F:	TGGGACWACATTTGGGAAGC	R:	TGGGATCAAACCACAAGTGA	113
MDP0000180902	F:	TGGAGCACAAAGTGGAACAA	R:	TGTATGGCTCGCAAATCTCA	155
MDP0000181339	F:	CCTGTTGGACATCAAGTTCG	R:	ATTGGAGACGGTTTTCTTGG	146
MDP0000196394	F:	CGCCGATCAAATGGTTCT	R:	TCTCTTCCCCTTCCTTCTCC	134
MDP0000197472	F:	AGGCTCAAGCATTTKGGTGT	R:	AGATTTTGGGGGTGAAGTGA	134
MDP0000204381	F:	TGTTGTCACGGGGTCYAT	R:	CGGCAAAAGGGTAAACGAC	163
MDP0000204699	F:	TGGYTRTACGGATGCTAGTGC	R:	CCTCTGAGTYGTAAACCAAAACATC	146
MDP0000204794	F:	AAAGCTGTGGCTGACGTTCT	R:	CTCCCTTCCCTTGATTGGTT	144
MDP0000205617	F:	CATGCCCGTAAGAAACAAGAA	R:	TTCACTTGCGAAATGGTACG	147
MDP0000206461	F:	GGTTGGCWGCGTCATAGAG	R:	TGTGGCTAGAAAGCGAGTCA	148
MDP0000211981	F:	GCTTGGGAGGAKYACTTTAAC	R:	CAGTCCCGAAYGTGAGAGTT	160
MDP0000212178	F:	GGAATCATCGRCTTTTCG	R:	TTTCTTTCTCCTCCTCCCATC	171
MDP0000219522	F:	CTTCCAATATCCCTTGCTGCT	R:	TCATGGTGCTGAACCTGCT	132
MDP0000219684	F:	GSKCRGATTTCGCATTTG	R:	CCGCTTGTCCAGGTTACTGT	160
MDP0000225509	F:	YAATYAGGCACAAGAACATCA	R:	TCCCAATCTGCTCCTCCYT	139
MDP0000232616	F:	TTTTGGGAATCCAACAGGAC	R:	GGCAGATGCAGCATAATTGA	154
MDP0000233546	F:	GCGTTGCCCACTTATCAAAC	R:	CGTCCAAGACCGATTCCAT	102

MDP0000236390	F:	GCCTCAATCCTCCTCACACT	R:	GAGACCGAYTGGGAAYACA	157
MDP0000236723	F:	ACCCGCTAAAYATCACCACATC	R:	KCTTGGGAGCAAAAAYACCR	153
MDP0000250070	F:	ATCAGCTTCTTGCCGAGTTC	R:	CATGGGGGCAGAACAATTA	115
MDP0000262141	F:	ACTGGTGGTCACGCTCTCTT	R:	TCCTCAAAGTAGTGCCTCTTCC	152
MDP0000264060	F:	ATCTGCCTCCGCACTCTATC	R:	CAGCTTCGTCCCCTTGAA	150
MDP0000264668	F:	TGGTGATGGTCGTGGAGTT	R:	TCTCATCCCTCCTTGCTCTC	116
MDP0000265729	F:	CAGCAGGCSMTGATGAAA	R:	CGAAAAYGGAGAGAGACGRAG	175
MDP0000265874	F:	GATTCCAGGAACTGGTGGTG	R:	GTCCATCATCCCCCTTTCTT	145
MDP0000268523	F:	GCACCCCAGCTGAAGTAGAG	R:	CGTAGGGCCAGTTCATTGTT	150
MDP0000272542	F:	ATGGACGGAGGATTRATGG	R:	TGCTGCTGGGCACTTTCYA	172
MDP0000277718	F:	AAAGGGTCRTCGGCTCTTG	R:	GCCATCCTCATCGTCTCAAT	143
MDP0000286136	F:	GCCAAATACGCAAGATAGCC	R:	TCTCCACCAACACCTTAGTCG	162
MDP0000289300	F:	GGAAAGCCYAGAGATGC	R:	GGGAAATCAAGCAGCACAAT	160
MDP0000294096	F:	GGCAGTCATCTCRGMACCT	R:	MRYACTCCACCAATCAAGTAACG	102
MDP0000296339	F:	CACCGAAGAAAGCGAAGAAT	R:	TGGCAAGGAGTGAGTTGATG	170
MDP0000307705	F:	AAGTTCCACCAGTCCCACAG	R:	GCTCCGCAAAGAAAAAGTTG	174
MDP0000309976	F:	TGTTAGTGGTGTCGCTTGGA	R:	ATTGGTTGCTCGGGATTGT	143
MDP0000311359	F:	TATGCAAGRATTGGGAAAGC	R:	TCCTCAGCTCCGTATTGTCC	157
MDP0000316497	F:	GAYGACAGCMGCATAAAAG	R:	ATTTCCYCCAATCCTCAGYT	112
MDP0000317974	F:	GCTGTGCTTRTCACTCTGCT	R:	ATGAAGAGAACGCCMARGAA	185
MDP0000320910	F:	CGGGACTGGAACACCTTCT	R:	GAAYGGGCAGTTSTGGWTGT	147
MDP0000324831	F:	TCCAAAACTAAGCCCCTCCT	R:	TCCAGACTCCTCCTTCATCG	150
MDP0000327191	F:	ATGGCGAAACCAAGTTCATC	R:	CTTGGGGTGGTAAGTCTCCA	151
MDP0000343634	F:	CAGGAGATCCAGGAAAAGGAC	R:	CAGTTCTGAAACAAGCCACAAC	112
MDP0000353793	F:	ATTATTACCCCACGCcCATT	R:	ATTACGCTGACCGaGAGATGTT	100
MDP0000364885	F:	GGGATTAGTGGTGGAGTTTA	R:	GGCACGTTACATGTCTTCATT	147
MDP0000404331	F:	TTGGTGATGGAAGTGGAGATT	R:	GCYTTGATGAGTAAYGGGTTTT	121
MDP0000440654	F:	TGAAGGCATGCCAGAAAGTT	R:	CTCCAGTTGGATTCGTCGTAA	111
MDP0000551952	F:	CCCTCTTTGGATTTGCCTAA	R:	ACGCTGGATTTACGGATTGT	163
MDP0000597996	F:	GGGGGTTCCAATCGTAATG	R:	CACGAAAGTGCATACCATGATT	158
MDP0000609876	F:	CARCAGATGACACCgCAATC	R:	CTSCCTCCAGCATTATTTCC	170
MDP0000609966	F:	MTTCTTCCCGTTCCATCGT	R:	TTTCTRTWCTGGTCGTAGAGGGATG	171
MDP0000612660	F:	CTCAACCTCAAGCCAATGTG	R:	CAACTTCGASATCAGCAAGG	154
MDP0000617684	F:	CCACCGAGGGCTAATCAKC	R:	ATGGAARTGGTCTCYGGGCTR	133
MDP0000628976	F:	TTCSGACGAAGAGAAAATCC	R:	ACACAGCGTCCTCCATATCTC	152
MDP0000644109	F:	TCCTTCAACTTCTGCCCCKA	R:	CGCTCCKRATTTCCTTCAAC	137
MDP0000668657	F:	CATTTGGGAATGCTGTGAACT	R:	GTGCAGCCAATCTTCATTAGG	136
MDP0000680997	F:	GTGCCAAACAAAGATGATGC	R:	CGAATGRGACCGARTGAGTG	178
MDP0000696168	F:	TTCTYAATGACACTGGGRTTCA	R:	GGAGGACGAGTGCTTGATTT	144
MDP0000711911	F:	CCTGTCCTGAACCCATCTGT	R:	GGGCGTCAGCAAGAAAGTAA	160
MDP0000737128	F:	AGRAGGGCRAGGTGGAAGAC	R:	GAGCCTGAAGRGCATCATT	100
MDP0000750217	F:	GGGCAGGATGAGTTTATTGG	R:	CGAGCCTTAGCGAGTCTATTG	131
MDP0000750789	F:	TTAAATTGCCTCCACACAAGC	R:	AATGGCTTTTCTTCCACAACC	163
MDP0000782642	F:	GAGCAGTTCCACCAGCAAG	R:	CAACAGACACGGGCAAGTT	108
MDP0000784187	F:	CAAAGTCCACACCCCAATCT	R:	AGGCTTSCCTCCGTTTTCT	144

MDP0000858763	F:	TTCCACTTTAYGAYGACAGCA	R:	TAYTTTCCGTTCCCCATTTG	196
MDP0000868044	F:	GGCACACTTGGTCACGATTA	R:	GCCTGRGAGCTTCTCATTTA	162
MDP0000874252	F:	GTCGTTTCGGTCCTCAAAAA	R:	AATCGGCAAATAATCGTCSA	162
MDP0000891117	F:	TCTRGGATAYCTYGGTGAGC	R:	GCCGAGGAGCTTCAWCTGT	154
MDP0000919962	F:	GGAGCAAGAGACGATCAAGG	R:	TTGAGAACACGCATGAGGAC	117
MDP0000921319	F:	AAGGGCTTGYTGAACTTGAA	R:	CCAGGAGGAGGAYTCTTGC	130
MDP0000929055	F:	CAGCTCCTCTGCAATTTATGG	R:	GGCGGTCGTCGTAACAATA	151
MDP0000937986	F:	CACTCCTCTGCCTTCTTTGG	R:	TCAGCGACATTGGTTTTCTG	153
MDP0000940742	F:	CACCACCAACTTCTTGCTCA	R:	GCTCAAACCAGTGCGACA	109
MDP0000944210	F:	TGAAWATTGGGACTACTGYTCA	R:	TGTCCTGRTGGGCAAGAT	114

[1]wobbles: M: A or C, R: A or G, W: A or T, S: G or C, Y: C or T, K: G or T
